# Association between CSF alpha-synuclein seeding activity and genetic status in Parkinson’s disease and dementia with Lewy bodies

**DOI:** 10.1186/s40478-021-01276-6

**Published:** 2021-10-30

**Authors:** Kathrin Brockmann, Corinne Quadalti, Stefanie Lerche, Marcello Rossi, Isabel Wurster, Simone Baiardi, Benjamin Roeben, Angela Mammana, Milan Zimmermann, Ann-Kathrin Hauser, Christian Deuschle, Claudia Schulte, Katharina Waniek, Ingolf Lachmann, Simon Sjödin, Ann Brinkmalm, Kaj Blennow, Henrik Zetterberg, Thomas Gasser, Piero Parchi

**Affiliations:** 1 Department of Neurodegeneration, Center of Neurology, Hertie Institute for Clinical Brain Research, German Center for Neurodegenerative Diseases, University of Tuebingen, Hoppe Seyler-Strasse 3, 72076 Tuebingen, Germany; 2 German Center for Neurodegenerative Diseases, University of Tuebingen, Tuebingen, Germany; 3 IRCCS Istituto delle Scienze Neurologiche di Bologna, Via Altura 1/8, 40139 Bologna, Italy; 4 Department of Experimental, Diagnostic and Specialty Medicine (DIMES), University of Bologna, Bologna, Italy; 5 Roboscreen GmbH, Leipzig, Germany; 6 Department of Psychiatry and Neurochemistry, Institute of Neuroscience and Physiology, The Sahlgrenska Academy at the University of Gothenburg, Mölndal, Sweden; 7 Clinical Neurochemistry Laboratory, Sahlgrenska University Hospital, Mölndal, Sweden; 8 Department of Neurodegenerative Disease, UCL Institute of Neurology, Queen Square, London, UK; 9 UK Dementia Research Institute at UCL, London, UK

**Keywords:** α-Syn seeding, RT-QuIC, CSF, PD, GBA, Parkin

## Abstract

The clinicopathological heterogeneity in Lewy-body diseases (LBD) highlights the need for pathology-driven biomarkers *in-vivo*. Misfolded alpha-synuclein (α-Syn) is a lead candidate based on its crucial role in disease pathophysiology. Real-time quaking-induced conversion (RT-QuIC) analysis of CSF has recently shown high sensitivity and specificity for the detection of misfolded α-Syn in patients with Parkinson's disease (PD) and dementia with Lewy bodies (DLB). In this study we performed the CSF RT-QuIC assay in 236 PD and 49 DLB patients enriched for different genetic forms with mutations in *GBA*, *parkin*, *PINK1*, *DJ1,* and *LRRK2*. A subgroup of 100 PD patients was also analysed longitudinally. We correlated kinetic seeding parameters of RT-QuIC with genetic status and CSF protein levels of molecular pathways linked to α-Syn proteostasis. Overall, 85% of PD and 86% of DLB patients showed positive RT-QuIC α-Syn seeding activity. Seeding profiles were significantly associated with mutation status across the spectrum of genetic LBD. In PD patients, we detected positive α-Syn seeding in 93% of patients carrying severe *GBA* mutations, in 78% with LRRK2 mutations, in 59% carrying heterozygous mutations in recessive genes, and in none of those with bi-allelic mutations in recessive genes. Among PD patients, those with severe *GBA* mutations showed the highest seeding activity based on RT-QuIC kinetic parameters and the highest proportion of samples with 4 out of 4 positive replicates. In DLB patients, 100% with *GBA* mutations showed positive α-Syn seeding compared to 79% of wildtype DLB. Moreover, we found an association between α-Syn seeding activity and reduced CSF levels of proteins linked to α-Syn proteostasis, specifically lysosome-associated membrane glycoprotein 2 and neurosecretory protein VGF.

These findings highlight the value of α-Syn seeding activity as an *in-vivo* marker of Lewy-body pathology and support its use for patient stratification in clinical trials targeting α-Syn.

**Supplementary Information:**

The online version contains supplementary material available at 10.1186/s40478-021-01276-6.

## Introduction

The current gold-standard diagnosis for Parkinson’s disease (PD) is based on clinicopathological criteria that include neuronal loss in the substantia nigra pars compacta and Lewy-body (LB) pathology related to alpha-synuclein (α-Syn) aggregation [1–3]. However, histopathological findings in some genetic forms of PD challenge this definition. While PD patients with mutations in the *GBA* gene (PD_GBA_) show extensive LB pathology, most PD patients with bi-allelic mutations in the recessive gene *parkin* (PD_recessive_bi-allelic_) show nigral degeneration without LBs [4]. The histopathology in PD patients with *LRRK2* mutations (PD_LRRK2_) is also remarkably variable, including typical LB pathology, misfolded tau deposition or nigral degeneration without LBs [5–7]. This heterogeneity highlights the need for pathology-driven biomarkers that distinguish between the different underlying pathologies *in-vivo*. With disease-modifying treatment options targeting α-Syn underway, patient stratification according to α-Syn-specific enrichment strategies is a much-needed prerequisite to introduce patients to clinical trials.

As we have no reliable imaging marker to assess the cerebral load of α-Syn *in-vivo*, research has focused on CSF. Yet, it is unclear whether CSF profiles of α-Syn species reflect α-Syn brain pathology. Analyses in sporadic PD and PD_GBA_ demonstrated decreased CSF levels of total α-Syn compared to controls with the highest decrease in PD_GBA_ patients carrying severe mutations [8–10]. However, a substantial inter-individual variability and overlap with controls are seen so that CSF levels of total α-Syn are not ideal as a single biomarker. Recently, the ultrasensitive assays real‐time quaking‐induced conversion (RT‐QuIC) and protein misfolding cyclic amplification have been successfully implemented. These assays exploit the seeding capacities of prion or prion-like proteins as an amplification strategy to reveal minute amounts of disease-specific protein aggregates in CSF [11, 12]. Both methods showed a high sensitivity of 88–96% and 83–98% specificity for sporadic LBD such as PD and DLB compared to controls [13, 14]. However, only one study on a limited number of patients has applied the α-Syn RT‐QuIC assay to patients with genetic PD [15].

## Materials and methods

### Aim, design and setting of the study

To further asses the value of α-Syn RT‐QuIC, as pathology-driven biomarker for LBD, we assessed CSF α-Syn seeding activity in two large cohorts with LBD enriched for genetic forms. We included PD and DLB patients with *GBA* mutations as a proxy for pronounced α-Syn pathology, and PD patients with *parkin*, *PINK1* and *DJ1* mutations as representatives for nigral degeneration with sparse or no α-Syn aggregation. Moreover, PD patients with *LRRK2* mutations were assessed. As no study has yet analyzed α-Syn seeding capacities longitudinally, we also evaluated α-Syn seeding profiles throughout the disease course in repeatedly collected CSF samples.

Besides, as several molecular pathways associated with α-Syn proteostasis have been identified over the last decade [16], we evaluated associations between α-Syn seeding profiles and CSF levels of proteins representative of these pathways.

### Participants

Between 2005 and 2020, 236 PD patients, 49 DLB patients, 14 asymptomatic mutation carriers, and 26 healthy controls have been recruited at the University Hospital of Tuebingen. Specifically, CSF of 108 sporadic PD patients (PD_wildtype_), 99 PD patients with *GBA* mutation (PD_GBA_), 9 PD patients with *LRRK2* mutation (PD_LRRK2_), 20 PD patients with mutations in *parkin, PINK1, or DJ1* (17 PD_recessive _heterozygous_, 3 PD_recessive_bi-allelic_), 33 DLB patients without *GBA* mutation (DLB_sporadic_) and 16 DLB patients with *GBA* mutation (DLB_GBA_) was available. See Additional file 1: Table S1 for the complete list of mutations.

Repeated lumbar punctures allowing longitudinal CSF measurements were performed in 100 PD patients (61 PD_wildtype_, 34 PD_GBA_, 2 PD_LRRK2_, 3 PD_recessive _heterozygous_). See Additional file 2: Figure S1.

### Genetic analysis

Genetic screening for mutations in *GBA, LRRK2, parkin*, *PINK1* and *DJ1* was done as previously described [17].

### Clinical investigations

All participants were examined by a movement disorders specialist. Diagnosis of PD was defined according to UK Brain Bank Society Criteria [18]. Diagnosis of DLB was made according to the DLB consortium revised consensus criteria; only patients with probable DLB were included [19]. Patients were assessed in dopaminergic ON. We assessed severity of motor symptoms using part III of the Unified Parkinson’s disease Rating Scale (UPDRS-III), from 2006–2008 the old version, from 2009 on the MDS-UPDRS [20]. Disease stage was categorized by modified Hoehn and Yahr Scale (H&Y) [21]. Cognitive function was tested with Montreal Cognitive Assessment (MoCA) [22] and/or Mini Mental Status Examination (MMSE) [23]. Since the MoCA was available only from 2009 on, previously obtained MMSE scores were converted into MoCA equivalents [24].

### CSF collection

Spinal tap was performed between 9.00 am and 1.00 pm. Samples were centrifuged within 60 min and frozen at − 80 °C within 90 min after collection. Samples with abnormal routine CSF diagnostics (erythrocytes > 1/µl, white blood cell count > 5 cells/µl, immunoglobulin subtype G index > 0.7) were excluded.

### Alpha-synuclein real-time quaking-induced conversion assay (RT-QuIC)

We performed purification of recombinant wildtype human α-Syn and the various steps of the RT-QuIC assay as described [14]*.* In addition to the negative control, we ran the same positive sample throughout all experiments to optimize the comparison between fluorescent responses in different plates. To overcome batch-to-batch variations and intrinsic plate-to-plate variability, we normalized the relative fluorescent units (RFU) for every time point to the maximum intensity reached by the positive control within each plate and expressed it as percentage. Intra-assay and inter-assays CVs before and after normalization are reported in Additional file 3: Table S2.

We calculated the threshold as the average normalized fluorescence value of negative control repeats during the first 10 h of recording, plus 30 standard deviations. The cut-off was set at 30 h. When only one of the four replicates crossed the threshold, the analysis was considered "unclear" and repeated up to three times. In those participants who showed a positive RT-QuIC α-Syn seeding profile (at least 2 out of 4 runs), we measured the area under the curve (AUC), the peak of the fluorescence response (Imax), and the lag phase (LAG) (time required to reach the threshold).

RT-QuIC experiments were performed at the Institute of Neurological Science of Bologna (ISNB). Results were reported blinded of the clinical diagnosis and genetic status. The assay showed a high specificity (98.7%) for LB pathology in a series of 121 CSF samples from individuals referred to the laboratory of neuropathology at ISNB for dementia of various etiologies in which the presence of LBs and abnormal α-Syn deposits was excluded by neuropathological examination [25].

### CSF measurement of total alpha-synuclein

CSF levels of total α-Syn were assessed using an ELISA kit for human α-Syn (Roboscreen GmbH, Germany). Intra-assay imprecision of 4.4% was calculated from duplicate analyses and expressed as median of the range to average of the duplicates. Inter-assay imprecision of < 10% was determined using two quality control CSF pool samples.

### CSF measurements of proteins related to alpha-synuclein proteostasis

Measurements of the following protein concentrations were performed using parallel reaction monitoring mass spectrometry (PRM-MS) as previously described [26, 27].

1. *Neurotransmitter secretion:* chromogranin-A (CHGA), secretogranin-2 (SCG2), neurosecretory protein VGF (VGF).
2. *Synapse plasticity:* neuronal pentraxin-1 (NPTX1).
3. *Autophagy, including endocytosis, lysosomal function, and ubiquitin–proteasome system:* AP-2 complex subunit beta (AP2B1), cathepsin F (CTSF), ganglioside GM2 activator (GM2A), lysosome-associated membrane glycoprotein 2 (LAMP2), ubiquitin.

#### Sample preparation

50μL CSF was mixed with 50μL of an internal standard mixture containing stable isotope-labelled peptides (JPT Peptide Technologies GmbH, Berlin, Germany; Thermo Fisher Scientific Inc. Waltham, MA, USA), 13C-labelled ubiquitin (Silantes, GmbH, Munich, Germany) and bovine serum albumin (Sigma-Aldrich Co., Saint Louis, MO, USA), diluted in 50 mM NH4HCO3. Reduction and alkylation was performed by addition of 50μL 15 mM 1,4-dithiothreitol in 50 mM NH4HCO3, shaking for 30 min at 60 °C, cooling down at room temperature for 30 min, and addition of 25μL 70 mM iodoacetamide in 50 mM NH4HCO3 followed by shaking at room temperature in the dark for 30 min. Samples were digested by the addition of 25μL 0.08 μg/μL sequencing grade modified trypsin (Promega Co., Madison, WI, USA) diluted in 50 mM NH4HCO3 and incubated at 37 °C shaking for 18 h. Digestion was ended by addition of 25μL 10% trifluoroacetic acid. Solid-phase extraction was performed using Oasis® HLB 96-well μElution Plates (2 mg sorbent and 30 μm particle size; Waters Co., Milford, MA, USA) by conditioning (2 × 300μL methanol), equilibration (2 × 300μL H2O), loading of samples, washing (2 × 300μL H2O), and elution (2 × 100μL methanol). Samples were dried by vacuum centrifugation and stored at − 80 °C.

#### Parallel reaction monitoring mass spectrometry (PRM-MS)

Samples were dissolved by addition of 50μL 50 mM NH4HCO3 and shaken at room temperature for 1 h. Forty μL of sample were injected and separated using a Dionex™ UltiMate™ 3000 standard-LC system (Thermo Fisher Scientific Inc., Waltham, MA, USA) and a Kinetex® EVO C18 column (length 150 mm; inner diameter 2.1 mm; particle size 1.7 μm; Phenomenex Inc., Torrance, CA, USA) with a SecurityGuard™ ULTRA cartridge prefilter (Phenomenex Inc.). On a 60 min method, with solvents A (0.1% formic acid in H2O (v/v)) and B (84% acetonitrile and 0.1% formic acid in H2O (v/v)), using a flow rate of 300μL/min, the gradient went from 3 to 5% B over one minute followed by 5–26% B over 48 min. The column temperature was set to 50 °C. Separation by high-performance liquid chromatography was performed in online mode coupled to a Q Exactive™ Hybrid Quadrupole-Orbitrap™ mass spectrometer (Thermo Fisher Scientific Inc.). Using a HESI-II ionization probe (Thermo Fisher Scientific Inc.) electrospray ionization was performed in positive ion mode with the following settings: spray voltage + 4.1 kV, heater temperature 400 °C, capillary transfer tube temperature 380 °C, sheath gas flow rate 25, auxiliary gas flow rate 10, and S-Lens RF level 60. Acquisition of data was performed using single microscan in PRM mode with an isolation window of m/z 2 centred on the second isotope of the precursor ion. The resolution setting was 70 k with an AGC target of 1 × 106 and a 256 ms injection time. Fragmentation was performed using higher energy collision-induced dissociation.

#### Data extraction

Skyline v.19.1 was used to calculate and export fragment ion peak areas and to monitor fragment ion traces and ratios. The ratio between tryptic peptide and isotope-labelled peptide peak area was used for quantification.

### CSF measurement of Aβ_1-42_, total-Tau (t-Tau), phospho-Tau (p-Tau), neurofilament light protein (NFL)

CSF levels of Aβ_1-42_, t-Tau and p-Tau were measured using ELISA kits from INNOTEST, Fujirebio GmbH, Germany. CSF levels of NFL were measured using the UmanDiagnostics NF-light®assay. Intra-assay coefficients of variation for each CSF parameter were below 15%.

#### Statistical analysis

Statistical analysis was performed using IBM SPSS 26.0 software. Group comparisons of dichotomous data were analyzed using the likelihood-ratio chi-square test as it is suited for unequally distributed samples sizes. Inter-group comparisons (disease groups stratified by genetic mutation; α-Syn seeders vs. non-seeders) of demographics, clinical markers, and CSF parameters were calculated using ANOVA/ANCOVA including sex, age, age at onset, and disease duration as co-variates as appropriate. Pearson’s correlation was used to evaluate associations between RT-QuIC seeding profiles and clinical markers and CSF parameters. Longitudinal analysis of RT-QuIC α-Syn seeding was done using linear mixed model with fixed factors *group* (seeders vs. non-seeders) and *time* (time of follow-up in years), their *interaction* and the random variable *subject*, modelled by random intercepts. Hypothesis testing was 2-sided and *p* values ≤ 0.05 were considered statistically significant.

## Results

Demographic and clinical characteristics of all groups and subgroups are given in Table 1 and 2 and Additional file 4: Table S3, Additional file 5: Table S4, and Additional file 6: Tables S5.

**Table 1:** RT-QuIC seeding profiles stratified by diagnosis

	PD n = 235	DLB n = 49	*p* Value PD/DLB vs. controls	Controls n = 26	Asymptomatic mutation carriers n = 14	*p* Value asymptomatic mutation carriers vs. Controls
Male Sex %	65	70	0.237	54	36	0.271
Age (y)	64 ± 9	72 ± 7***	≤ 0.001	59 ± 12	58 ± 15	0.855
Age at onset (y)	57 ± 10	68 ± 7***	–	–	–	–
Disease duration (y)	7 ± 6	3 ± 2***	–	–	–	–
UPDRS III	25 ± 11	30 ± 13*	≤ 0.001	2 ± 2	1 ± 1	0.112
MoCA	25 ± 4	15 ± 6***	≤ 0.001	27 ± 3	28 ± 2	0.303
LEDD	544 ± 475	392 ± 216*	–	–	–	
RT-QuIC positive seeding n (%)	200 (85)	42 (86)	≤ 0.001	2 (8)	2 (14)	0.516
RT-QuIC 0/4 positive seeding n (%)	35 (15)	7 (14)	≤ 0.001	18 (92)	12 (86)	0.363
RT-QuIC 2/4 positive seeding n (%)	18 (8)	1 (2)		1 (4)	0	
RT-QuIC 3/4 positive seeding n (%)	54 (23)	12 (25)		0	1 (7)	
RT-QuIC 4/4 positive seeding n (%)	128 (54)	29 (59)		1 (4)	1 (7)	
RT-QuIC AUC	761 ± 239	804 ± 225	–	–	–	–
RT-QuIC Imax	70 ± 13	69 ± 12	–	–	–	–
RT-QuIC LAG	21 ± 3	20 ± 3	–	–	–	–
CSF total alpha-synuclein pg/ml	567 ± 262	535 ± 322*	0.175	583 ± 181	627 ± 389	0.677
CSF Aβ_1–42_ pg/ml	713 ± 262	518 ± 229**	≤ 0.001	925 ± 231	885 ± 407	0.693
CSF t-Tau pg/ml	242 ± 132	328 ± 238	0.151	240 ± 97	242 ± 120	0.955
CSF p-Tau pg/ml	41 ± 16	49 ± 28	0.429	41 ± 13	44 ± 17	0.561
NFL pg/ml	917 ± 850	1921 ± 1888***	≤ 0.001	542 ± 239	431 ± 91	0.294

*MoCA* montreal cognitive assessment, *UPDRS III* Unified Parkinson Disease Rating Scale part III, *LEDD* L-Dopa equivalent daily dose

*p* values were corrected for age and sex when compared to controls and age, age at onset and disease duration for comparison of PD vs. DLB where appropriate. Significance level PD vs. DLB: *p < 0.05, **p < 0.01, ***p ≤ 0.001

**Table 2:** RT-QuIC seeding profiles in PD stratified by mutation status

	PD_wildtype_ n = 107	PD_GBA_risk_ n = 53	PD_GBA_mild_ n = 17	PD_GBA_severe_ n = 29	PD_LRRK2_ n = 9	PD_recessive_heterozygous_ n = 17	PD_recessive_bi-allelic_ n = 3	*p* value
Male sex %	70	70	65	66	33	41	33	0.096
Age (y)	65 ± 8	65 ± 9	66 ± 9	59 ± 10	65 ± 13	62 ± 13	63 ± 9	0.070
Age at onset (y)	61 ± 8	58 ± 10	57 ± 9	51 ± 10	53 ± 14	51 ± 13	36 ± 6	≤ 0.001
Disease Duration (y)	5 ± 3	8 ± 5	8 ± 6	9 ± 7	12 ± 7	11 ± 10	27 ± 2	≤ 0.001
UPDRS III	23 ± 10	28 ± 11	28 ± 13	27 ± 13	27 ± 9	27 ± 11	22 ± 1	0.265
MoCA	26 ± 3	24 ± 5	25 ± 6	24 ± 5	24 ± 4	26 ± 3	27 ± 5	0.005
LEDD	389 ± 259	595 ± 363	626 ± 252	701 ± 545	802 ± 399	830 ± 1241	1497 ± 1481	
RT-QuIC positive (%)	97 (91)	48 (91)	11 (65)	27 (93)	7 (78)	10 (59)	0 (0)	≤ 0.001
RT-QuIC 0/4 positive (%)	10 (9)	5 (9)	6 (35)	2 (7)	2 (22)	7 (41)	3 (100)	≤ 0.001
RT-QuIC 2/4 positive (%)	13 (12)	1 (2)	1 (6)	0 (0)	0 (0)	3 (18)	0 (0)	
RT-QuIC 3/4 positive (%)	29 (27)	11 (21)	3 (18)	5 (17)	3 (33)	3 (18)	0 (0)	
RT-QuIC 4/4 positive (%)	55 (52)	36 (68)	7 (41)	22 (76)	4 (45)	4 (23)	0 (0)	
RT-QuIC AUC	713 ± 238	795 ± 281	797 ± 157	858 ± 179	843 ± 191	702 ± 202	n.a	0.048
RT-QuIC Imax	68 ± 14	71 ± 15	72 ± 12	71 ± 10	75 ± 7	67 ± 12	n.a	0.378
RT-QuIC LAG	21 ± 3	20 ± 3	20 ± 2	19 ± 2	19 ± 3	21 ± 2	n.a	0.009
CSF total α-Syn pg/ml	578 ± 279	560 ± 212	489 ± 220	512 ± 273	712 ± 319	567 ± 221	840 ± 487	0.171
CSF Aβ_1–42_ pg/ml	679 ± 249	684 ± 268	706 ± 214	779 ± 248	972 ± 302	753 ± 299	854 ± 262	0.208
CSF t-Tau pg/ml	232 ± 127	265 ± 152	241 ± 100	203 ± 86	334 ± 190	261 ± 144	179 ± 19	0.191
CSF p-Tau pg/ml	41 ± 16	39 ± 12	43 ± 15	36 ± 16	54 ± 21	45 ± 20	42 ± 6	0.227
NFL pg/ml	894 ± 633	1042 ± 1320	1099 ± 965	839 ± 635	832 ± 311	720 ± 419	571 ± 87	0.816

*MoCA* Montreal cognitive assessment, *UPDRS III* Unified Parkinson Disease Rating Scale part III, *n.a.* not applicable, *LEDD* L-Dopa equivalent daily dose

*p* values were corrected for age, age at onset and disease duration

### RT-QuIC seeding activity is associated with disease status and genetic mutations

#### Association with disease

Eighty-five percent of PD and 86% of DLB patients showed a positive RT-QuIC α-Syn seeding activity compared to 8% of controls (Table 1). Of the asymptomatic mutation carriers, 14% (1 *GBA*, 1 *LRRK2)* were α-Syn seeding positive. A single PD patient showing 1 out of 4 positive replicates by RT-QuIC in three runs was excluded from analyses given the inconclusive result.

There were no significant differences in RT-QuIC seeding kinetics (AUC, Imax, and LAG) between PD and DLB patients with positive α-Syn seeding (*p* > 0.05).

Male sex and higher age were associated with positive α-Syn seeding (sex: r = 0.139, *p* = 0.019) and higher numbers of positive RT-QuIC replicates (sex: r =  − 0.133, *p* = 0.024; age: r = 0.127, *p* = 0.033).

#### Association with genetic mutations

**Fig. 1:**
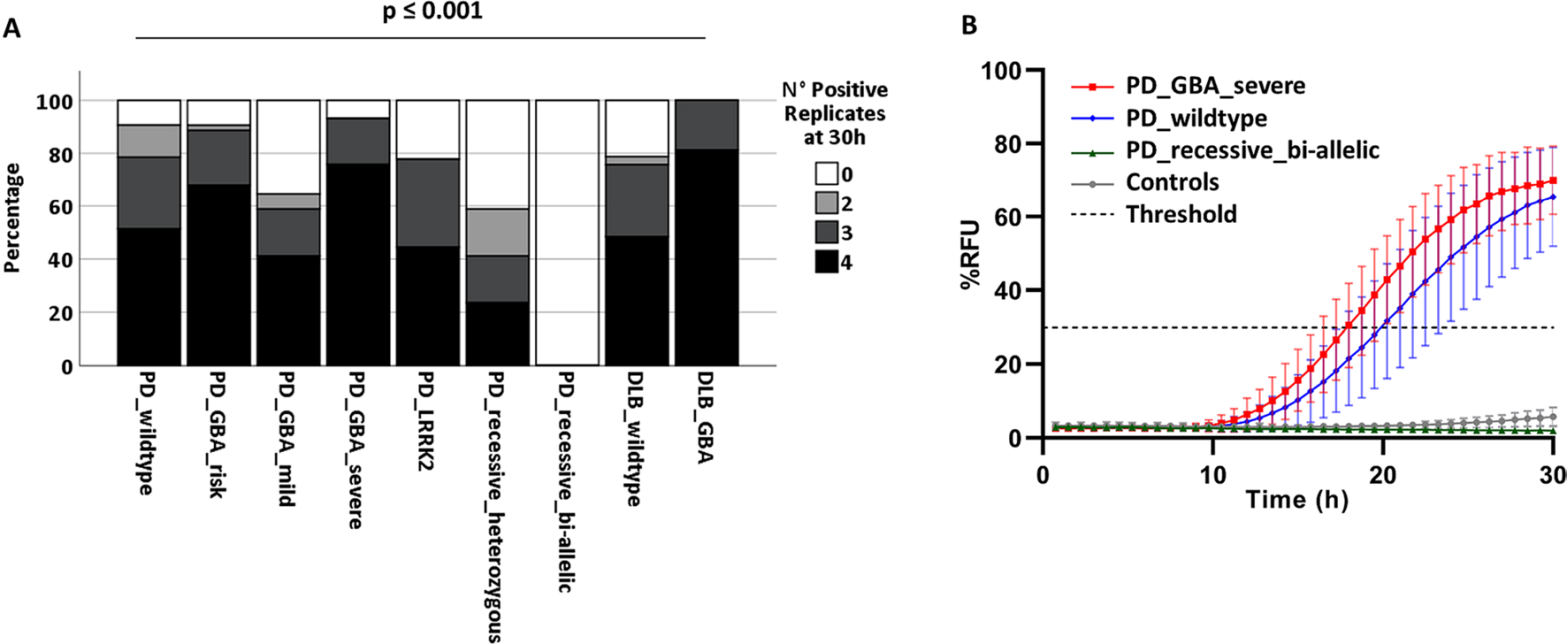
RT-QuIC positive replicates relative fluorescence curves at 30 h stratified by diagnosis and mutation status. **A** Remarkable differences in RT-QuIC α-Syn seeding profiles were detected in the PD patients’ group when stratifying by PD-associated mutations. While 93% of PD_GBA_severe_ showed a positive RT-QuIC α-Syn seeding profile, 78% of PD_LRRK2_ and only 59% of PD_recessive _heterozygous_ gave a positive reaction by RT-QuIC. Strikingly, none of the PD_recessive_bi-allelic_ showed a positive RT-QuIC α-Syn seeding profile (overall *p* ≤ 0.001) Numbers (n) included: PD_wildtype_ = 107, PD_GBA_risk_ = 53, PD_GBA_mild_ = 17, PD_GBA_severe_ = 29, PD_LRRK2_ = 9, PD_recessive_heterozygous_ = 17, PD_recessive_biallelic_ = 3, DLB_wildtype_ = 33, DLB_GBA_ = 16. **B** While PD_GBA_severe_ patients showed the strongest RT-QuIC seeding kinetics measured by the mean relative fluorescence (RFU) of positive curves (see comparison with PD_wildtype_), PD_recessive_bi-allelic_ did not seed at all.

While 91% of PD_wildtype_ and 87% of PD_GBA_ (93% of PD_GBA_severe_) showed positive α-Syn seeding activity, 78% of PD_LRRK2_ and only 59% of PD_recessive _heterozygous_ gave a positive reaction. None of the PD_recessive_bi-allelic_ showed positive α-Syn seeding (overall *p* ≤ 0.001) (Fig. 1A, B).

In PD patients with positive α-Syn seeding, AUC was highest in PD_GBA_severe_ (median 858 RFU) and lowest in PD_recessive _heterozygous_ (median 702 RFU; overall *p* = 0.048). Accordingly, PD_GBA_severe_ had the shortest LAG phase (19 h; overall *p* = 0.009) (Table 2).

PD_GBA_severe_ showed also the highest proportion of samples with 4 out 4 positive replicates among PD groups (overall *p* ≤ 0.001).

In DLB, 100% of DLB_GBA_ showed positive α-Syn seeding activity compared to 79% of DLB_wildtype_ (*p* = 0.014) (Fig. 1A).

AUC, Imax, and LAG in DLB patients with positive α-Syn seeding were comparable between wildtype and *GBA* status (*p* > 0.05, respectively) (Additional file 4: Table S3).

### RT-QuIC seeding activity is associated with lower CSF protein levels related to alpha-synuclein-proteostasis

Positive α-Syn seeding and higher numbers of positive replicates were associated with lower CSF levels of LAMP2 (r =  − 0.178, *p* = 0.012; r =  − 0.146, *p* = 0.040) and VGF (r =  − 0.187, *p* = 0.008; r =  − 0.160, *p* = 0.023).

Higher RT-QuIC AUC and shorter LAG were associated with lower CSF levels of AP2B1 (r =− 0.224, *p* = 0.003; r = 0.215, *p* = 0.004), CHGA (r =  − 0.176, *p* = 0.018; r = 0.168, *p* = 0.024), CTSF (r =  − 0.176, *p* = 0.018; r = 0.161, *p* = 0.030), SCG2 (r =  − 0.185, *p* = 0.012; r = 0.159, *p* = 0.033), ubiquitin (r =  − 0.219, *p* = 0.004; r = 0.239, *p* = 0.002), and VGF (r =  − 0.204, *p* = 0.006; r = 0.184, *p* = 0.013) (Additional file 5: Table S4).

Correspondingly, α-Syn seeders showed lower mean CSF levels of LAMP2 (PD: 1.00) and VGF (PD: 0.93; DLB: 0.57) compared to non-seeders (PD: LAMP2 1.27, *p* = 0.05, VGF 1.36, *p* = 0.014; DLB: VGF 0.90, *p* = 0.05) (Additional file 6: Table S5).

Higher age and female sex were associated with higher CSF levels of AP2B1, CHGA, GM2A, LAMP2, SCG2, ubiquitin, and VGF (*p* < 0.01, respectively). No associations were found with disease duration.

### RT-QuIC seeding activity is associated with motor and cognitive impairment

Positive α-Syn seeding and higher numbers of positive RT-QuIC replicates were associated with higher UPDRS-III (r = 0.148, *p* = 0.024; r = 0.189, *p* = 0.004) and lower MoCA scores (r =  − 0.163, *p* = 0.016; r =  − 0.256, *p* ≤ 0.001).

Higher RT-QuIC AUC and shorter LAG were associated with higher UPDRS-III (r = 0.150, *p* = 0.034; r =  − 0.165, *p* = 0.020) and lower MoCA scores (r =  − 0.189, *p* = 0.009; r = 0.186, *p* = 0.011) (Additional file 5: Table S4). There were no associations with disease duration.

### RT-QuIC alpha-synuclein seeders compared to non-seeders

Despite a shorter disease duration (7 years), PD patients with positive α-Syn seeding activity had a higher UPDRS-III (26) and lower MoCA score (25) compared to non-seeders (9 years disease duration, UPDRS-III 21, MoCA 27; *p* < 0.01 respectively).

DLB patients with positive α-Syn seeding activity had a higher prevalence of REM-sleep behaviour disorder (RBD) (50% vs. 0%, *p* = 0.049) whereas sex, age, age at onset, disease duration, prevalence of parkinsonism and the interval between onset of parkinsonism and onset of dementia were not statistically significant when compared to non-seeders. DLB RT-QuIC non-seeders had higher mean CSF levels of t-Tau (533 vs. 294; *p* = 0.012) and NFL (3586 vs. 1630; *p* = 0.010) compared to RT-QuIC seeders (Additional file 6: Table S5).

### RT-QuIC seeding profiles remain stable over time

**Fig. 2:**
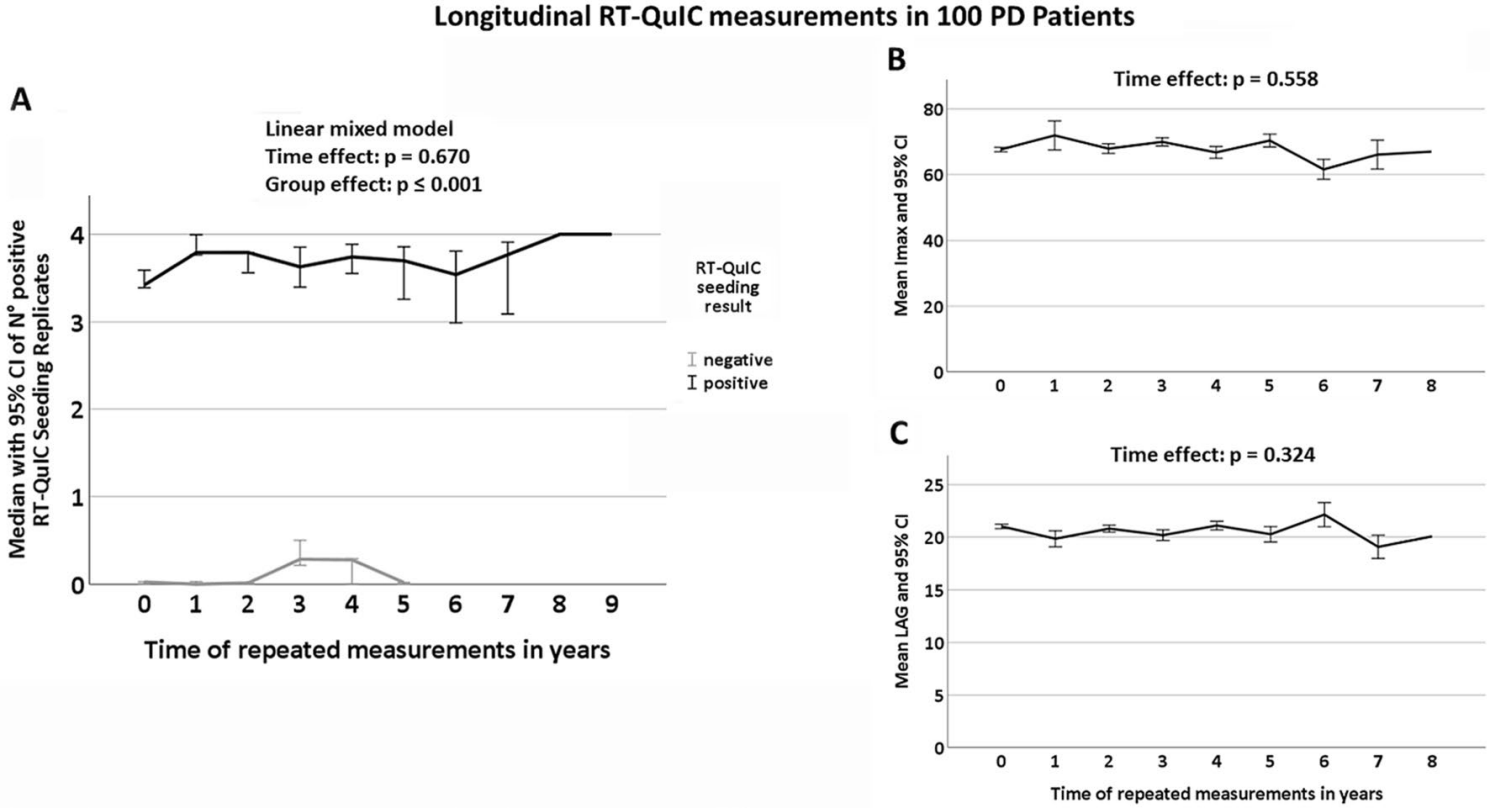
Longitudinal trajectories of RT-QuIC seeding profiles. Out of the 100 PD patients included in this longitudinal analysis, 86 were RT-QuIC seeding positive while 14 did not show seeding. **A** Linear mixed model analysis in the longitudinal PD cohort with repeated lumbar puncture and RT-QuIC assay measurements revealed that *time* had no significant impact on the number of positive RT-QuIC seeding replicates over the course of the disease, neither in those patients who were seeding positive nor in those who were seeding negative (*p* = 0.670). **B, C** Similarly, RT-QuIC seeding positives showed stable results of Imax (*p* = 0.558) and LAG phase (*p* = 0.324) over time

Out of 100 PD patients included in this longitudinal analysis, 86 were RT-QuIC seeding positive. Mean time between first and last lumbar puncture was 3.5 years in RT-QuIC seeding positive and 3.4 years in RT-QuIC seeding negative patients. Linear mixed model analyses revealed that the number of positive RT-QuIC replicates did not change over time, neither in the overall longitudinal PD cohort (*p* = 0.670) (Fig. 2A) nor in those PD patients with first LP at disease duration < 2 years (*p* = 0.949). Similarly, α-Syn seeding positives showed stable results of AUC (*p* = 0.576), Imax (*p* = 0.558) and LAG phase (*p* = 0.324) over time (Fig. 2B, C).

## Discussion

The present results show a significant association between RT-QuIC α-Syn seeding activity and mutation status across the LBD spectrum. Remarkably, mutations in *GBA*, especially those classified as severe, showed the highest percentage of positive α-Syn seeding activity in both PD (93%) and DLB (100%). In contrast, PD patients with bi-allelic mutations in recessively inherited genes like *parkin* or *PINK1* did not show CSF α-Syn seeding at all, whereas those carrying heterozygous mutations in these genes showed less α-Syn seeding than wild-type cases with a reduced positivity rate of 59%. Also, PD patients with *LRRK2* mutations showed a reduced rate of α-Syn seeding (78%), although not to the extent previously reported in a Spanish cohort carrying the p.G2019S mutation [15]. The broader spectrum of mutations in our cohort, along with differences in clinical features such as older age and longer disease duration, might have contributed to these different results. Larger cohorts of patients carrying other mutations are needed to comprehensively evaluate the effect of *LRRK2* on α-Syn pathology.

Overall, 85% of PD and 86% of probable DLB patients showed α-Syn seeding activity, which is slightly lower than the percentage obtained in previous studies [11–13]. The characteristics of our cohort, enriched for different genetic mutations, including those not harbouring LB pathology, best explain the results in PD patients. In contrast, the relatively low number of patients combined with the purely clinical diagnosis (akinetic-rigid parkinsonism, dementia and hallucinations in all cases) without supportive biomarkers (MIBG, DaTScan) possibly explain the lower frequency of positive cases in our wild-type DLB cohort. Moreover, previously analyzed DLB cohorts might have included some patients carrying a mutation in *GBA* given that they were not characterized genetically. Interestingly, patients with DLB that were RT-QuIC seeding negative had fewer clinical core features related to α-Syn pathology such as RBD [28]. These results suggest that most of the negative α-Syn RT-QuIC outcomes we obtained in patients with a clinical diagnosis of DLB did not indicate false-negative assay results. Indeed, it is well known from post-mortem and CSF studies that heterogeneous pathologies may sustain this clinical syndrome [19]. Consistent with this interpretation is the finding that DLB patients carrying *GBA* mutations, who were all positive by α-Syn RT-QuIC, showed normal mean CSF profile of t-Tau, p-Tau and Aβ_1–42,_ whereas the group of sporadic DLB patients without *GBA* mutations presented higher mean CSF levels of t-Tau, p-Tau and NfL as well as reduced Aβ_1–42_ levels indicating a typical Alzheimer’s disease profile. The heterogeneity in α-Syn seeding activity among the different genetic forms of PD mirrors histopathological findings in these cases. While PD patients with *GBA* mutations show extensive α-Syn-positive LB pathology, those with bi-allelic mutations in *parkin* or *PINK1* show nigral degeneration without LB, whereas those carrying *LRRK2* mutations show LB pathology in most cases, but also misfolded tau deposition or nigral degeneration without LB pathology [4, 29].

Additionally, we demonstrated an association between RT-QuIC parameters expressing higher α-Syn seeding activity (higher AUC, shorter LAG) with lower CSF levels of proteins related to α-Syn proteostasis, including autophagy, endocytosis, lysosomal function, ubiquitin–proteasome system and neurosecretion. Accordingly, PD and DLB patients with positive RT-QuIC seeding showed reduced CSF levels of lysosomal-associated membrane glycoprotein 2 and neurosecretory protein VGF compared to patients with negative seeding. Recent studies reported lower CSF protein levels of AP-2-complex subunit beta, cathepsin F, ganglioside GM2 activator, lysosomal-associated membrane glycoprotein 1 and 2, and ubiquitin in PD compared to patients with Alzheimer’s disease and controls, and decreased CSF levels of chromogranin-A and B, neurosecretory protein VGF, and secretogranin species in PD and DLB compared to controls [30, 31]. Interestingly, lower CSF levels of VGF were associated with lower CSF levels of α-Syn while no association was found with Aβ_1–42_ [32, 33]. These data suggest that amplification assays such as RT-QuIC might provide new tools to study *in-vivo* the mechanistic link between proteins linked to α-Syn proteostasis and seeding, which was previously shown only in post-mortem studies and cell models [34–39].

Besides the capacity to accurately discriminate patients with LB pathology *in-vivo*, it will be essential to determine whether α-Syn RT-QuIC may have a role in predicting clinical trajectories. Increased α-Syn seeding capacities might predispose to accelerated/widespread α-Syn aggregation, thereby promoting faster progression to clinical disease milestones such as cognitive impairment. Our finding of an association between RT-QuIC kinetics parameters (AUC, LAG) with more severe motor impairment and cognitive dysfunction suggests that this might be the case. However, additional studies in prospective longitudinal cohorts where CSF and clinical data are available in de-novo PD patients with several years of follow-up, combined with further refinement of the quantitative capabilities of the assay are needed to fully address the value of the α-Syn RT-QuIC as a prognostic marker in LBD patients.

Most asymptomatic mutation carriers did not show RT-QuIC α-Syn seeding. The two RT-QuIC α-Syn positive mutation carriers (1 *GBA*, 1 *LRRK2*) were older and had lower MoCA scores than the negative ones. The observation is in line with a recent study in *LRRK2* mutation carriers where only 19% showed a positive seeding compared to 20% of the control group. Those with positive seeding activity had higher likelihood ratios for probable prodromal PD [15]. These findings warrant further longitudinal investigations of larger cohorts with mutation carriers enriched for prodromal symptoms specific for α-Syn such as RBD.

Repeated longitudinal measurements revealed stable RT-QuIC α-Syn seeding profiles. This finding implicates that seeding activity represents a trait marker for α-Syn pathology rather than a marker that simply increases with time or disease duration. Further analysis in longitudinal cohorts with de-novo patients is needed to evaluate α-Syn seeding activity in different disease stages prospectively. The full reproducibility of the α-Syn RT-QuIC positive vs. negative outcome in samples taken from the same patients longitudinally also supports the assay's value as a specific pathology-driven biomarker of α-Syn pathology.

Strengths of our study include the large monocentric standardized collection of CSF samples minimizing variance in sample collection and processing, the validation of findings in longitudinal samples from the same patients, and the reproducibility in two alpha-synucleinopathies.

Limitations are the lack of longitudinal correlation between CSF RT-QuIC α-Syn seeding activity and post-mortem brain histopathology and the small/imbalanced sample sizes of genetic subgroups and those stratified by RT-QuIC seeding profiles.

## Conclusions

Our finding of heterogeneous seeding profiles across genetic forms of LBD suggest that CSF RT-QuIC α-Syn seeding activity is highly representative of and mirrors LB brain pathology *in-vivo*. We also show that RT-QuIC α-Syn seeding activity is associated with reduced CSF levels of proteins related to α-Syn proteostasis supporting mechanistic links suggested by in-vitro models. Importantly, this assay allows patient stratification according to α-Syn-specific enrichment strategies in preparation for clinical trials targeting α-Syn.

## Supplementary Information

**Additional file 1: Table S1.** Genetic mutations stratified by gene in PD and DLB. The table shows the prevalence of genetic mutations observed in each group of the analysed cohort.

**Additional file 2: Figure S1.** Schematic representation of study design. Between 2005 and 2020, 236 PD patients, 49 DLB patients, 14 asymptomatic mutation carriers, and 26 healthy controls have been recruited at the University Hospital of Tuebingen. Specifically, CSF of 108 sporadic PD patients (PD_wildtype_), 99 PD patients with GBA mutation (PD_GBA_), 9 PD patients with LRRK2 mutation (PD_LRRK2_), 20 PD patients with mutations in parkin, PINK1, or DJ1 (17 PD_recessive _heterozygous_, 3 PD_recessive_bi-allelic_), 33 DLB patients without GBA mutation (DL_Bsporadic_) and 16 DLB patients with GBA mutation (DL_BGBA_) was available. Repeated lumbar punctures allowing longitudinal CSF measurements were performed in 100 PD patients (61 PDwildtype, 34 P_DGBA_, 2 PD_LRRK2_, 3 PD_recessive _heterozygous_).

**Aditional file 3: Table S2.** Intra-batch and inter-batch (overall) coefficients of variation (%) of quantitative RT-QuIC parameters Imax and AUC of the positive control, before (raw) and after normalization. The intra-batch coefficients of variation (CV) of the maximum intensity of fluorescence (I max) and area under the curve (AUC) are expressed as percentage of the ratio between standard deviation and average.

**Additional file 4: Table S3.** RT-QuIC seeding profiles in DLB stratified by GBA mutation status. The table shows the α-syn seeding profiles of the analysed DLB group (number of positive replicates, area under the curve, Imax and LAG) together with the quantification of CSF Aβ1-42, t-Tau, p-Tau and NFL (all measures are expressed in pg/ml).3.Additional file 3: Table S3 (.txt). RT-QuIC seeding profiles in DLB stratified by GBA mutation status. The table shows the α-syn seeding profiles of the analysed DLB group (number of positive replicates, area under the curve, Imax and LAG) together with the quantification of CSF Aβ1-42, t-Tau, p-Tau and NFL (all measures are expressed in pg/ml).

**Aditional file 5: Table S4**. Correlations between CSF RT-QuIC alpha-synuclein seeding parameters with clinical measures and with CSF protein levels related to alpha-synuclein proteostasis in PD and DLB. RT-QuIC alpha-synuclein seeding activity is associated with higher motor impairment as measured by the Unified Parkinson Disease Rating Scale III (UPDRS III) and lower cognitive function measured by Montreal Cognitive Assessment (MoCA). Moreover, higher RT-QuIC alpha-synuclein seeding capacity is associated with lower CSF levels of proteins that are linked to lysosomal dysfunction and neurotransmitter secretion.

**Additional file 6: Table S5.** Clinical and CSF characteristics of RT-QuIC seeders vs. non-seeders. p-Value significantly different between RT-QuIC alpha-synuclein seeders vs. non-seeders in the respective diagnostic groups.

## Data Availability

Anonymized data are available upon reasonable request to: kathrin.brockmann@uni-tuebingen.de.

## Abbreviations

ISNB: Institute of Neurological Science of Bologna
LP: Lumbar puncture
LBD: Lewy body disease
LB: Lewy body
CSF: Cerebrospinal fluid
α-Syn: Alpha-synuclein
RT-QuIC: Real-time quaking-induced conversion
PD: Parkinson's disease
DLB: Dementia with Lewy bodies
PD_GBA_: PD patients with mutations in the *GBA* gene, further classified as PD_GBA_risk_, PD_GBA_mild_ or PD_GBA_severe_ based on the specific mutation/s occurring in the *GBA* gene
PD_recessive_bi-allelic_: PD patients with bi-allelic mutations in the recessive genes *parkin*, *PINK1 or DJ1*
PD_recessive _heterozygous_: PD patients with mutations in *parkin, PINK1*, or *DJ1*
PD_LRRK2_: PD patients with *LRRK2* mutations
PD_wildtype_: Patients with sporadic PD
DLB_sporadic_: Patients with sporadic DLB
DLB_GBA_: DLB patients with *GBA* mutations
MoCA: Montreal cognitive assessment
MMSE: Mini mental status examination
AUC: Area under the curve
Imax: Maximum of intensity (related to RT-QuIC)
LAG: The lag phase (time required to reach the threshold)
PRM-MS: Parallel reaction monitoring mass spectrometry
CHGA: Chromogranin-A
SCG2: Secretogranin-2
VGF: Neurosecretory protein VGF
NPTX1: Neuronal pentraxin-1
AP2B1: AP-2 complex subunit beta
CTSF: Cathepsin F
GM2A: Ganglioside GM2 activator
LAMP2: Lysosome-associated membrane glycoprotein 2
Aβ_1–42_: Beta-amyloid protein, fragment 1–42
t-Tau: Total Tau
p-Tau: Tau phosphorylated at position 181
NFL: Neurofilament light chain
MIBG: Nuclear medicine imaging test using metaiodobenzylguanidine
DaTScan: Dopamine active transfer scan
