# Supplementary material for: Association between CSF alpha-synuclein seeding activity and genetic status in Parkinson’s disease and dementia with Lewy bodies
Source: Acta Neuropathol Commun. 2021 Oct 30;9:175. doi: 10.1186/s40478-021-01276-6 (PMC8556894; doi:10.1186/s40478-021-01276-6)
Supplement: Supplementary file 1 — Additional file 1: Table S1. Genetic mutations stratified by gene in PD and DLB. The table shows the prevalence of genetic mutations observed in each group of the analysed cohort. [file 40478_2021_1276_MOESM1_ESM.docx]

**Table S1**

**Genetic mutations stratified by gene in PD and DLB.**

|  | PD_GBA_risk_  n=53 | PD_GBA_mild_  n=17 | PD_GBA_severe_  n=29 | PD_LRRK2_  n=9 | PD_recessive_heterozygous_  n=17 | PD_recessive_bi-allelic_  n=3 | DLB_GBA_  n=16 |
| --- | --- | --- | --- | --- | --- | --- | --- |
| Mutation  n (%) | E326K; 34 (68)  T369M; 12 (28)  R39C; 1 (2)  T297S; 1 (2) | N370S; 14 (94)  S271G+L268L; 1 (6) | L444P; 12 (41)  IVS2+1 g>a; 3 (10)  L444P+A456P+V640V; 2 (7)  W184R; 2 (7)  c.1265-1319del55bp  +D409H+L444P+A456P+V460V; 1(3)  D409H; 1 (3)  G202R; 1 (3)  H255Q 1 (3)  L444P+E326K+A456P+V640V; 1 (3)  L444P+E326K+N392; 1 (3)  L444P+E388K; 1 (3)  L444P+T369M; 1 (3)  R359X; 1 (3)  Y304C+I2020T (LRKK2); 1 (3) | G2019S; 4 (44)  N1437S; 2 (22)  R1441C; 2 (22)  I2020T; 1 (11) | **PINK1:**  G411S; 1 (6)  **PRKN:**  P437L; 4 (24)  R275W; 4 (24)  R234Q; 1 (6)  R256C; 1 (6)  c.101-102delAG+ex3-4del; 1 (6)  ex2del+ex7dup; 1 (6)  ex2del; 1 (6)  ex3-4del; 1 (6)  ex8-9del; 1 (6)  **DJ1:**  ex4-5dup; 1 (6) | **PINK1:**  Q126P; 2 (66)  **PRKN:**  ex3-6del; 1 (33) | E326K; 5 (32)  T369M; 4 (25)  N370S; 2 (13)  L444P; 1 (6)  ex3+4rec; 1(6)  L444P, A456P, V460V, c.*92g>a, c.*102t>c; 1 (6)  P182L; 1 (6)  R120Q; 1 (6) |
