# Supplementary figures and images for: Association between CSF alpha-synuclein seeding activity and genetic status in Parkinson’s disease and dementia with Lewy bodies
Source: Acta Neuropathol Commun. 2021 Oct 30;9:175. doi: 10.1186/s40478-021-01276-6 (PMC8556894; doi:10.1186/s40478-021-01276-6)

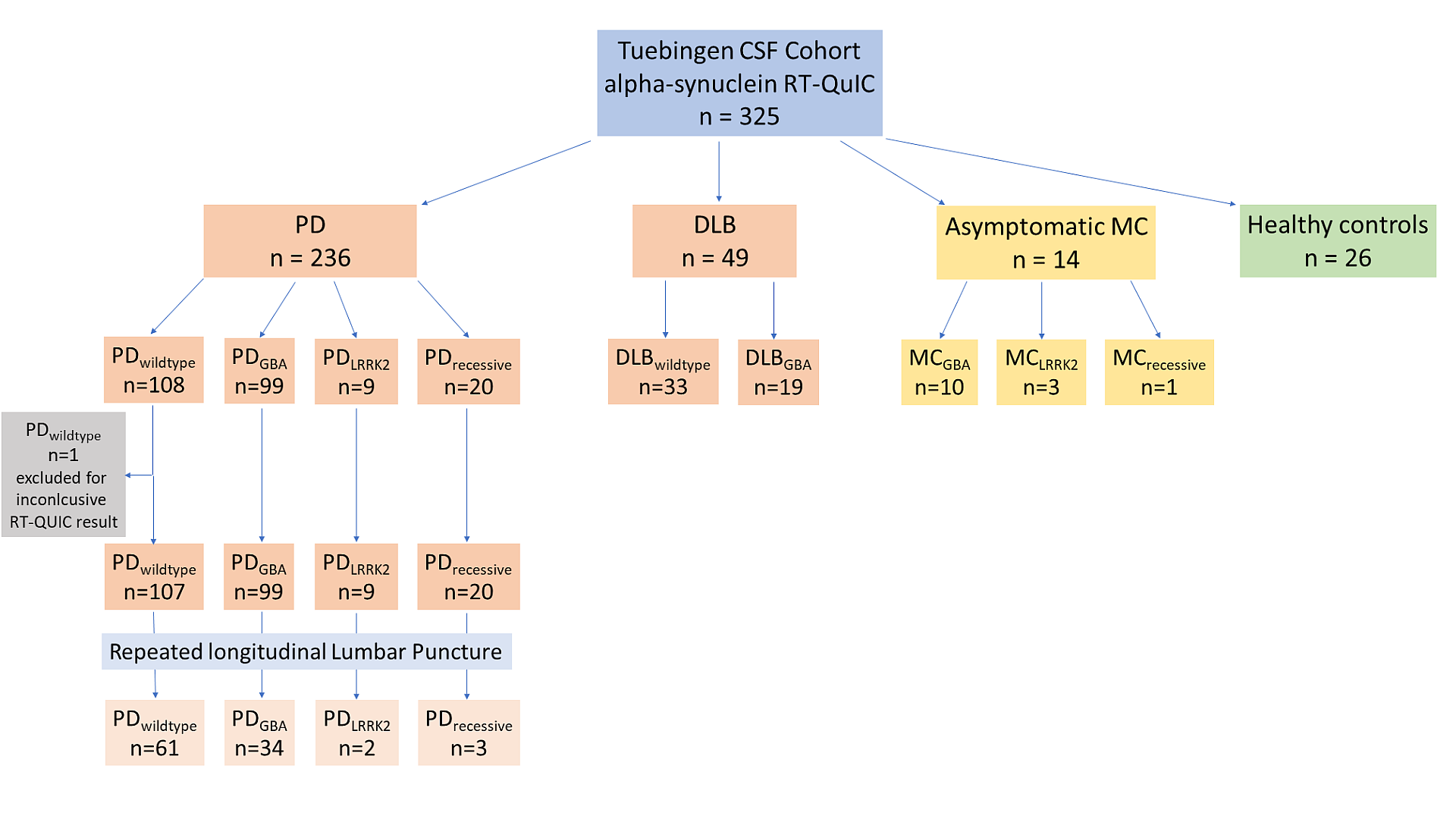

Supplement: Supplementary file 2 — Additional file 2: Figure S1. Schematic representation of study design. Between 2005 and 2020, 236 PD patients, 49 DLB patients, 14 asymptomatic mutation carriers, and 26 healthy controls have been recruited at the University Hospital of Tuebingen. Specifically, CSF of 108 sporadic PD patients (PDwildtype), 99 PD patients with GBA mutation (PDGBA), 9 PD patients with LRRK2 mutation (PDLRRK2), 20 PD patients with mutations in parkin, PINK1, or DJ1 (17 PDrecessive _heterozygous, 3 PDrecessive_bi-allelic), 33 DLB patients without GBA mutation (DLBsporadic) and 16 DLB patients with GBA mutation (DLBGBA) was available. Repeated lumbar punctures allowing longitudinal CSF measurements were performed in 100 PD patients (61 PDwildtype, 34 PDGBA, 2 PDLRRK2, 3 PDrecessive _heterozygous). [file 40478_2021_1276_MOESM2_ESM.tif]
