# Supplementary material for: Association between CSF alpha-synuclein seeding activity and genetic status in Parkinson’s disease and dementia with Lewy bodies
Source: Acta Neuropathol Commun. 2021 Oct 30;9:175. doi: 10.1186/s40478-021-01276-6 (PMC8556894; doi:10.1186/s40478-021-01276-6)
Supplement: Supplementary file 3 — Aditional file 3: Table S2. Intra-batch and inter-batch (overall) coefficients of variation (%) of quantitative RT-QuIC parameters Imax and AUC of the positive control, before (raw) and after normalization. The intra-batch coefficients of variation (CV) of the maximum intensity of fluorescence (I max) and area under the curve (AUC) are expressed as percentage of the ratio between standard deviation and average. [file 40478_2021_1276_MOESM3_ESM.docx]

**Table S2**

**Intra-batch and inter-batch (overall) coefficients of variation (%) of quantitative RT-QuIC parameters Imax and AUC of the positive control, before (raw) and after normalization.**

|  |  | **Imax** | | **AUC** | |
| --- | --- | --- | --- | --- | --- |
|  | **α-syn**  **Batch n°** | **Norm CVs %** | **Raw CVs %** | **Norm CVs %** | **Raw CVs %** |
| **Positive control** | 1  2  3  4  5  6  7 | 4,7  5,8  7,2  9,8  6,0  7,1  2,5 | 9,2  18,5  20,1  24,8  22,1  34,6  37,1 | 10,4  9,8  7,7  7,8  16,8  12,3  19,2 | 14,9  21,7  26,9  26,1  32,5  42,8  52,2 |
|  | **Overall CV %** | **6,8** | **22,3** | **11,8** | **26,0** |

The intra-batch coefficients of variation (CV) of the maximum intensity of fluorescence (I max) and area under the curve (AUC) are expressed as percentage of the ratio between standard deviation and average.
