# Supplementary material for: Association between CSF alpha-synuclein seeding activity and genetic status in Parkinson’s disease and dementia with Lewy bodies
Source: Acta Neuropathol Commun. 2021 Oct 30;9:175. doi: 10.1186/s40478-021-01276-6 (PMC8556894; doi:10.1186/s40478-021-01276-6)
Supplement: Supplementary file 4 — Additional file 4: Table S3. RT-QuIC seeding profiles in DLB stratified by GBA mutation status. The table shows the α-syn seeding profiles of the analysed DLB group (number of positive replicates, area under the curve, Imax and LAG) together with the quantification of CSF Aβ1-42, t-Tau, p-Tau and NFL (all measures are expressed in pg/ml).3.Additional file 3: Table S3 (.txt). RT-QuIC seeding profiles in DLB stratified by GBA mutation status. The table shows the α-syn seeding profiles of the analysed DLB group (number of positive replicates, area under the curve, Imax and LAG) together with the quantification of CSF Aβ1-42, t-Tau, p-Tau and NFL (all measures are expressed in pg/ml). [file 40478_2021_1276_MOESM4_ESM.docx]

**Table S3**

**RT-QuIC seeding profiles in DLB stratified by *GBA* mutation status.**

|  | DLB_wildtype_  n=33 | DLB_GBA_  n=16 | p-Value |
| --- | --- | --- | --- |
| Male Sex % | 63 | 84 | 0.079 |
| Age (y) | 73 ± 7 | 70 ± 5 | 0.107 |
| Age at onset (y) | 69 ± 8 | 66 ± 6 | 0.154 |
| Disease Duration (y) | 3 ± 2 | 3 ± 1 | 0.879 |
| UPDRS III | 33 ± 18 | 28 ± 8 | 0.676 |
| MoCA | 15 ± 4 | 15 ± 7 | 0.958 |
| LEDD | 300 ± 239 | 381 ± 158 |  |
| RT-QuIC positive seeding n (%) | 26 (79) | 16 (100) | 0.014 |
| RT-QuIC 0/4 positive seeding n (%) | 7 (21) | 0 (0) | 0.036 |
| RT-QuIC 2/4 positive seeding n (%) | 1 (3) | 0 (0) |  |
| RT-QuIC 3/4 positive seeding n (%) | 9 (27) | 3 (19) |  |
| RT-QuIC 4/4 positive seeding n (%) | 16 (49) | 13 (81) |  |
| RT-QuIC AUC | 825 ± 235 | 771 ± 210 | 0.463 |
| RT-QuIC Imax | 70 ± 13 | 67 ± 11 | 0.536 |
| RT-QuIC LAG | 20 ± 3 | 20 ± 3 | 0.604 |
| CSF total alpha-synuclein pg/ml | 578 ± 339 | 445 ± 273 | 0.175 |
| CSF Aβ_1-_42 pg/ml | 479 ± 226 | 599 ± 221 | 0.085 |
| CSF t-Tau pg/ml | 379 ± 264 | 223 ± 123 | 0.031 |
| CSF p-Tau pg/ml | 54 ± 31 | 37 ± 19 | 0.049 |
| NFL pg/ml | 2154 ± 2164 | 1424 ± 978 | 0.220 |

MoCA = Montreal cognitive assessment. UPDRS III = Unified Parkinson Disease Rating Scale part III.
