# Supplementary material for: Association between CSF alpha-synuclein seeding activity and genetic status in Parkinson’s disease and dementia with Lewy bodies
Source: Acta Neuropathol Commun. 2021 Oct 30;9:175. doi: 10.1186/s40478-021-01276-6 (PMC8556894; doi:10.1186/s40478-021-01276-6)
Supplement: Supplementary file 5 — Aditional file 5: Table S4. Correlations between CSF RT-QuIC alpha-synuclein seeding parameters with clinical measures and with CSF protein levels related to alpha-synuclein proteostasis in PD and DLB. RT-QuIC alpha-synuclein seeding activity is associated with higher motor impairment as measured by the Unified Parkinson Disease Rating Scale III (UPDRS III) and lower cognitive function measured by Montreal Cognitive Assessment (MoCA). Moreover, higher RT-QuIC alpha-synuclein seeding capacity is associated with lower CSF levels of proteins that are linked to lysosomal dysfunction and neurotransmitter secretion. [file 40478_2021_1276_MOESM5_ESM.docx]

**Table S4**

**Correlations between CSF RT-QuIC alpha-synuclein seeding parameters with clinical measures and with CSF protein levels related to alpha-synuclein proteostasis in PD and DLB.**

|  | RT-QuIC positive seeding | RT-QuIC N° positive replicates at 30h | RT-QuIC AUC | RT-QuIC Imax | RT-QuIC LAG |
| --- | --- | --- | --- | --- | --- |
| Age | 0.026 | 0.127* | 0.087 | 0.084 | -0.055 |
| Sex | -0.139* | -0.133* | 0.059 | 0.057 | -0.059 |
| Disease duration | -0.116 | -0.054 | 0.049 | 0.041 | -0.081 |
| UPDRS III | 0.148* | 0.189** | 0.150* | 0.098 | -0.165* |
| MoCA | -0.163* | -0.256*** | -0.189** | -0.153* | 0.186* |
| LEDD | -0.106 | -0.054 | -0.012 | -0.089 | -0.044 |
| AP2 complex subunit beta | -0.117 | -0.101 | -0.224** | -0.184* | 0.215** |
| Chromogranin A | -0.132 | -0.115 | -0.176* | -0.137 | 0.168* |
| Cathepsin F | -0.018 | -0.011 | -0.176* | -0.131 | 0.161* |
| Ganglioside GM2 activator | -0.111 | -0.082 | -0.176* | -0.157* | 0.146 |
| LAMP2 | -0.178* | -0.146* | 0.127 | 0.088 | -0.144 |
| Neuronal pentraxin 1 | -0.104 | -0.088 | -0.178* | -0.152* | 0.139 |
| Secretogranin 2 | -0.145* | -0.121 | -0.185* | -0.139 | 0.159* |
| Ubiquitin | -0.067 | -0.056 | -0.219** | -0.170* | 0.239** |
| Neurosecretory protein VGF | -0.187** | -0.160* | -0.204** | -0.145 | 0.184* |
| CSF total alpha-synuclein pg/ml | -0.104 | -0.074 | -0.122 | -0.086 | 0.105 |
| CSF Aβ_1-_42 pg/ml | -0.022 | -0.057 | -0.102 | -0.090 | 0.071 |
| CSF t-Tau pg/ml | -0.109 | -0.047 | -0.064 | -0.054 | 0.052 |
| CSF p-Tau pg/ml | -0.106 | -0.065 | -0.074 | -0.060 | 0.063 |
| NFL pg/ml | -0.107 | -0.065 | -0.105 | -0.094 | 0.099 |

MoCA = Montreal cognitive assessment. UPDRS III = Unified Parkinson Disease Rating Scale part III.

Significance level: * p<0.05, ** p<0.01, *** p≤0.001.

RT-QuIC alpha-synuclein seeding activity is associated with higher motor impairment as measured by the Unified Parkinson Disease Rating Scale III (UPDRS III) and lower cognitive function measured by Montreal Cognitive Assessment (MoCA). Moreover, higher RT-QuIC alpha-synuclein seeding capacity is associated with lower CSF levels of proteins that are linked to lysosomal dysfunction and neurotransmitter secretion.
