# Supplementary material for: Association between CSF alpha-synuclein seeding activity and genetic status in Parkinson’s disease and dementia with Lewy bodies
Source: Acta Neuropathol Commun. 2021 Oct 30;9:175. doi: 10.1186/s40478-021-01276-6 (PMC8556894; doi:10.1186/s40478-021-01276-6)
Supplement: Supplementary file 6 — Additional file 6: Table S5. Clinical and CSF characteristics of RT-QuIC seeders vs. non-seeders. p-Value significantly different between RT-QuIC alpha-synuclein seeders vs. non-seeders in the respective diagnostic groups. [file 40478_2021_1276_MOESM6_ESM.docx]

**Table S5**

**Clinical and CSF characteristics of RT-QuIC seeders vs. non-seeders.**

|  | **PD**  **n=235** | | **DLB**  **n=49** | |
| --- | --- | --- | --- | --- |
|  | RT-QuIC  positive  seeding  n=200 | RT-QuIC  negative  seeding  n=35 | RT-QuIC  positive  seeding  n=42 | RT-QuIC  negative  seeding  n=7 |
| Male Sex % | 68 | 51 | 74 | 43 |
| Age (y) | 64 ± 9 | 63 ± 10 | 72 ± 6 | 72 ± 9 |
| Age at onset (y) | 58 ± 9 | 55 ± 14 | 68 ± 7 | 68 ± 11 |
| Disease Duration (y) | 7 ± 5* | 9 ± 10 | 4 ± 2 | 3 ± 1 |
| UPDRS III | 26 ± 11** | 21 ± 10 | 30 ± 13  (n=19) | 35  (n=1) |
| MoCA | 25 ± 4** | 27 ± 2 | 14 ± 6  (n=16) | 19 ± 6  (n=2) |
| Parkinsonism (%) |  |  | 97 | 100 |
| Prevalence of RBD (%) |  |  | 50* | 0 |
| Interval Parkinsonism to Dementia (months) |  |  | 6 ± 6 | 9 ± 7 |
| AP2 complex subunit beta | 0.97 ± 0.37  (n=143) | 1.14 ± 0.68  (n=14) | 0.91 ± 0.45  (n=37) | 1.08 ± 0.67  (n=7) |
| Chromogranin A | 0.95 ± 0.62  (n=143) | 1.26 ± 0.97  (n=14) | 0.72 ± 0.48  (n=38) | 1.00 ± 0.91  (n=7) |
| Cathepsin F | 1.00 ± 0.25  (n=143) | 1.01 ± 0.31  (n=14) | 0.97 ± 0.26  (n=38) | 1.01 ± 0.46  (n=7) |
| Ganglioside GM2 activator | 0.98 ± 0.35  (n=143) | 1.10 ± 0.54  (n=14) | 0.93 ± 0.41  (n=38) | 1.13 ± 0.76  (n=7) |
| LAMP2 | 1.00 ± 0.45*  (n=142) | 1.27 ± 0.73  (n=14) | 0.91 ± 0.38  (n=37) | 1.23 ± 0.92  (n=7) |
| Neuronal pentraxin 1 | 1.01 ± 0.42  (n=142) | 1.19 ± 0.68  (n=14) | 0.78 ± 0.35  (n=38) | 0.96 ± 0.80  (n=7) |
| Secretogranin 2 | 0.94 ± 0.43  (n=143) | 1.18 ± 0.67  (n=14) | 0.74 ± 0.35  (n=38) | 0.98 ± 0.60  (n=7) |
| Ubiquitin | 0.96 ± 0.35  (n=137) | 1.01 ± 0.43  (n=14) | 1.01 ± 0.46  (n=35) | 1.15 ± 0.59  (n=7) |
| Neurosecretory protein VGF | 0.93 ± 0.56**  (n=143) | 1.36 ± 0.86  (n=14) | 0.57 ± 0.32*  (n=38) | 0.90 ± 0.74  (n=7) |
| CSF total alpha-synuclein pg/ml | 562 ± 259  (n=192) | 595 ± 285  (n=34) | 491 ± 299*  (n=42) | 796 ± 354  (n=7) |
| CSF Aβ_1-42_ pg/ml | 713 ± 262  (n=197) | 716 ± 266  (n=35) | 508 ± 228  (n=42) | 579 ± 241  (n=7) |
| CSF t-Tau pg/ml | 240 ± 131  (n=197) | 251 ± 138  (n=35) | 294 ± 188**  (n=42) | 533 ± 394  (n=7) |
| CSF p-Tau pg/ml | 40 ± 15  (n=194) | 44 ± 19  (n=35) | 47 ± 27  (n=39) | 60 ± 34  (n=7) |
| NFL pg/ml | 916 ± 850  (n=191) | 924 ± 864  (n=33) | 1630 ± 1494**  (n=40) | 3586 ± 3005  (n=7) |

MoCA = Montreal cognitive assessment. UPDRS III = Unified Parkinson Disease Rating Scale part III.

*p-Value significantly different between RT-QuIC alpha-synuclein seeders vs. non-seeders in the respective diagnostic groups. * p<0.05, ** p<0.01, *** p≤0.001.
